# Intrinsically fluorescent and quercetin loaded highly crosslinked polyphosphazene nanospheres: synthesis, characterization and fluorescence properties

**DOI:** 10.55730/1300-0527.3433

**Published:** 2022-04-19

**Authors:** Simge METİNOĞLU ÖRÜM

**Affiliations:** * Department of Chemistry, Faculty of Science and Art, Burdur Mehmet Akif Ersoy University, Burdur, Turkey

**Keywords:** Fluorescence, phosphazene, highly crosslinked, inorganic-organic hybrid, fluorescein, quercetin

## Abstract

Highly crosslinked, inorganic-organic hybrid and intrinsically fluorescent polyphosphazene nanospheres bearing hydroxyl groups on the surface are facilely generated via a one-pot polycondensation of octachlorocyclotetraphosphazene, fluorescein and quercetin. The resulting nanospheres were characterised by scanning electron microscopy (SEM), energy-dispersive X-ray analysis (EDX), Fourier transform infrared spectroscopy (FTIR), dynamic light scattering (DLS), X-ray diffraction analysis (XRD) and ultraviolet-visible spectroscopy (UV-vis) techniques. The average diameter of the nanospheres was determined as 379 nm. Also, quercetin which is both a monomer and an anticancer drug was loaded to the nanospheres as 446 mg g^−^^1^. The obtained nanospheres possess outstanding disperse ability in both aqueous and organic solvents. Moreover, the nanospheres exhibited intrinsically fluorescence intensity and outstanding photobleaching stability under ultraviolet-visible irradiation, due to the highly crosslinked and inorganic-organic hybrid structure. Owing to these superior properties and novelty of synthesized nanospheres, they have a great potential in many applications such as fluorescent labels, sensors, cell imaging and as a nanocarrier of quercetin for cancer treatment.

## 1. Introduction

Fluorescence technology, owing to its high sensitivity, efficiency and ease operation, [1,2] has been widely used for sensors, [3,4] diagnostics, [5,6] biological imaging [7,8] and light emitting diodes [9,10]. A variety of fluorescent nanomaterials, for instance, semiconductor quantum dots (QDs) [11,12], multifunctional nanoshells [13,14], carbon dots [15,16], organic fluorescent dyes [17,18], and dye-doped nanoparticles [19,20] have been utilized for these applications. However, the applications of these fluorescent materials significantly restricted due to their toxic nature and poor chemical stability. For example, the organic fluorescent dyes have high quantum yields and fluorescence intensity. But, they have also limitations such as hydrophobicity, concentration-quenching effect and poor photostability in the presence of oxygen [21]. Because of this situation, the organic fluorescent dyes have encapsulated into nanoparticles [22,23], micelles [24] or vesicles [25]. Although the encapsulation technique efficiently improves the dispersion and emission properties of organic fluorescent dyes, leakage of them from host systems is an important problem, due to the organic fluorescent dyes are only physically participated [26]. Hence, the development of synthesis route of intrinsically fluorescent nanoparticles with outstanding properties such as high fluorescence emission and photostability, good dispersibility in organic and aqueous medium, and wide applications is still considered as an ongoing challenge.

Apart from the materials mentioned above, the polyphosphazenes have special properties including highly crosslinked structure[27], dispersibility in water and organic media [28], biodegradability [29], and biocompatibility [30]. Therefore, highly crosslinked inorganic-organic polyphosphazene nano/micromaterials have been synthesized via polycondensation polymerization between hexachlorocyclotriphosphazene (HCCP) [31–33] or octachlorocyclotetraphosphazene (OCCP) [34,35] and monomers including two or more amino/hydroxyl/thyol groups [36–38], and their fluorescence applications have investigated such as fluorescent sensors to detection of some metal ions (Fe^3+^, Hg^2+^) [39, 40], nitroaromatic explosives [41], picric acid [42], dopamine [43], drug delivery [44], cell [45,46], and tumour imaging [47].

Fluorescein is a type of xanthane dye with yellowish green fluorescence using as generally fluorescence probe for detect some metals such as copper, mercury, iron, palladium, cadmium and magnesium in aqueous solutions and living cells, and as fluorescent label. However, it has some disadvantages like all solvents cannot be used with it, and also its photobleaching property is not good [48]. Besides, some nanomaterials have been synthesized by encapsulation of fluorescein though its leakage from the host [49–51].

On the other hands, quercetin is a flavonoid compound and is widely found in vegetables, leaves and fruits. It has also anticancer, antiviral and antiinflammatory properties. However, it has a limited clinical use due to its poor water solubility and very low bioavailability [52]. Quercetin has been loaded to many kinds of materials such as cubosomes [53], nanomicelles [54], silica nanoparticles [55], nanoliposome [56], gelatin films [57], and its anticancer [58, 59], antioxidant properties [60, 61] and release profile [62, 63] have been studied.

In this work, novel highly crosslinked and intrinsically fluorescent polyphosphazene nanospheres were prepared via one-pot polycondensation using fluorescein, quercetin and OCCP as monomers and crosslinker, respectively. The leakage of fluorescein from the nanospheres prevented due to the monomers bounded to the crosslinked structure covalently by nucleophilic substitution. Also, quercetin was loaded to the nanospheres as an anticancer drug besides it is a monomer. The nanospheres exhibited well dispersity in aqueous and organic media, high fluorescence emission and photobelaching property without further modifications. The synthesized polyphosphazene nanospheres have a great potential for sensors, labels, carriers, owing to these outstanding properties. Besides, the prepared fluorescent nanospheres can be used for in vivo delivery of quercetin, and cell imaging.

## 2. Materials and methods

Octachlorocyclotetraphosphazene (OCCP) was used after recrystallized with dry n-heptane. Quercetin, fluorescein, triethylamine (TEA), acetone and ethanol were purchased from Sigma-Aldrich and were used without purification.

Scanning electron microscopy (SEM–EDX) analysis was made on a ZEISS GeminiSEM 500 electron microscope at an accelerating voltage of 3 kV. Perkin Elmer FTIR Spectrometer Spotlight 400 Imaging System was used for Fourier transform infrared spectroscopy (FTIR) measurements of the nanospheres, OCCP, quercetin and fluorescein. X-ray diffraction (XRD) pattern was recorded by using a Bruker AXS, D8 Advance instrument equipped with Cu Kα radiation at 40 kV and 40 mA. The UV-vis measurements were recorded by the PG Instruments T60 Model UV-vis spectrophotometer. The fluorescence experiments were performed using the LS-55 Fluorescence Spectrometers. Solid-state ^31^P and ^1^H-NMR spectra were recorded by JEOL ECZ500R Spectrometer operating at 500 MHz.

### 2.1. Synthesis of the nanospheres and loading quercetin

OCCP (0.1 g; 0.216 mmol), quercetin (0.065 g; 0.216 mmol) and fluorescein (0.072 g; 0.216 mmol) were dissolved in 50 mL acetone, under sonication (53 kHz, 150 W) for 15 min. Then, triethylamine (3 mL, TEA) was added slowly to the reaction. After 4 h, the reaction medium was centrifuged at 4500 rpm, for 10 min and yellow product was collected. Finally, the obtained polyphosphazene nanospheres were washed with acetone, distilled water and ethanol, respectively. They were dried under vacuum at 50 °C. The reaction yield was 60%.

The amount of loaded quercetin in the nanospheres was calculated by UV-vis measurements using calibration curve obtained with standard quercetin solutions (Figure S1). After the reaction finished, the absorbance of the filtrate was measured at 372 nm to determine amount of unreacted quercetin. The quantity of the loaded quercetin in the nanospheres DL (%) could be calculated from the difference between the unreacted quercetin and the totally feeding quercetin, Equation (1). Besides, entrapment efficiency EE(%) of quercetin was calculated by Equation (2).


(1) $$\text{DL}(\%)=[{\text{M}}_{\text{Q}}-{\text{M}}_{\text{unQ}}]/{\text{M}}_{\text{nanospheres}}]\times 100$$

(2) $$\text{EE}(\%)=[({\text{Total}}_{\text{Q}}-{\text{Residual}}_{\text{Q}})/{\text{Total}}_{\text{Q}}]\times 100$$

M_nanospheres_, M_Q_ and M_unQ_ are the mass of the nanospheres, the total quercetin and the unreacted quercetin, respectively.

## 3. Results and discussion

### 3.1. Preparation of nanospheres and loading quercetin

**Figure 1:**
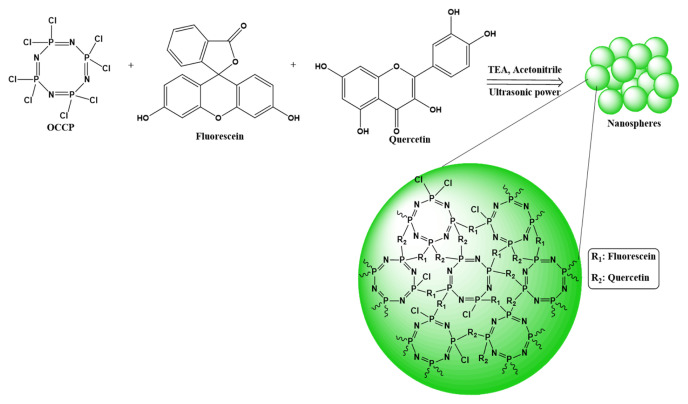
The reaction pathway of the polyphosphazene nanospheres.

The cyclomatrix type, highly crosslinked, intrinsically fluorescent polyphosphazene nanospheres were synthesized via a one-pot polycondensation polymerization technique. Two monomers (quercetin and fluorescein) and a crosslinker (OCCP) were used in the reaction. The hydroxyl groups on the fluorescein and quercetin were activated by excess TEA which is an acid acceptor to absorb the obtaining HCl. The TEA.HCl salts were formed during the reaction and polymerization were accelerated [64]. The nanospheres were prepared without further modification at one step, just 4 h using ultrasonic power. The reaction pathway and opened crosslinked structure of the nanospheres can be seen in Figure 1. The nanospheres are formed by self-assembly polycondensation polymerization. In this mechanism, oligomeric species are formed when quercetin, fluorescein and OCCP reacted in the presence of TEA at first. Then, the primary nucleus particles generate by aggregation of the oligomers. After, the primary nucleus particles aggregate together by hydrogen bonds and the stable particles are formed. Finally, the highly crosslinked, inorganic-organic hybrid and solid polyphosphazene nanospheres are formed by absorbing oligomers and growing in size of the stable particles [65]. The formation mechanism of the nanospheres can be seen in Figure S2.

Quercetin was bonded to OCCP covalently by this synthesis mechanism. The drug loading DL (%) and entrapment efficiency EE (%) were calculated as 44.60% and 96.77%, respectively. Also, it was determined that the nanospheres contained 446 mg g^−^^1^ quercetin. As can be seen in Table, DL (%) and EE (%) of quercetin are higher comparing with many reports [66–72].

### 3.2. Characterization of nanospheres

**Figure 2:**
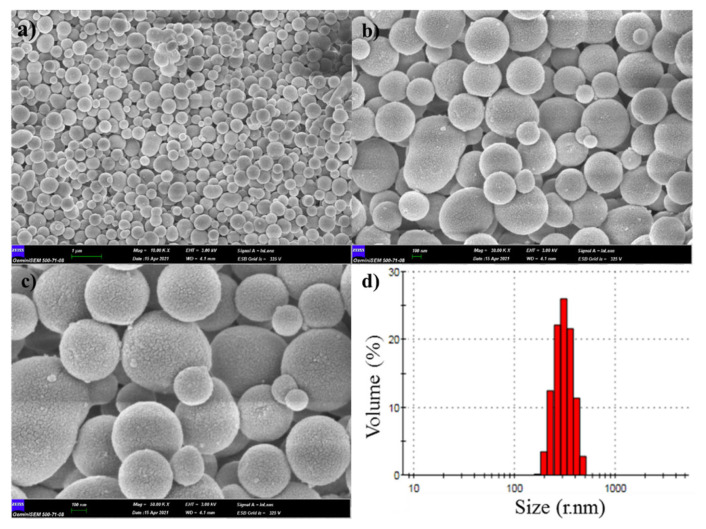
The SEM images of the nanospheres at different magnifications a) 10.00 KX, b) 30.00 KX, c) 50.00 KX, d) the particle size distribution of the nanospheres.

It was seen that the obtained nanospheres are spherical by the SEM images at different magnifications including 10.00, 30.00, and 50.00 KX in Figures 2a–2c. The average particle size and particle size distribution of the nanospheres were determined as 379 nm and 171–477 nm, respectively by dynamic light scattering (DLS) measurements (Figure 2d).

**Figure 3:**
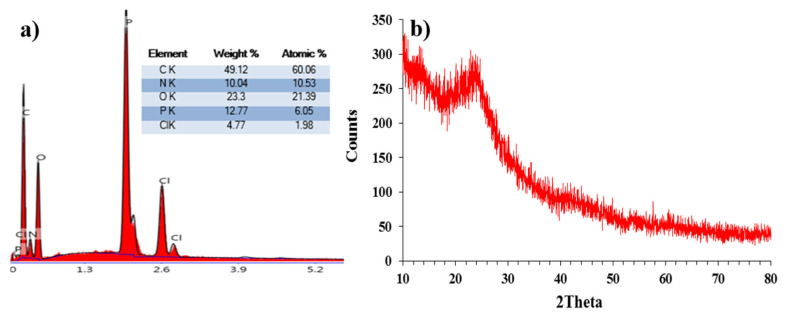
EDX results and XRD pattern of the nanospheres.

The EDX results of the nanospheres are shown in Figure 3a. It was determined that the nanospheres have 60.06% C and 21.39% O atoms which indicate including quercetin and fluorescein in their structures. Phosphorus and nitrogen atoms belonging to OCCP were identified as 6.05% and 10.53%, respectively. Because of the steric hindrance, the nanospheres have only unreacted 1.98% Cl atoms, demonstrated that the nanospheres are highly crosslinked.

The XRD pattern of the prepared nanospheres is shown in Figure 3b. The characteristic wide diffraction peak between 20 and 30 θ indicates that the nanospheres were amorphous without any crystallization and they were purified well from quercetin, fluorescein, OCCP and TEA.HCl salt [73].

**Figure 4:**
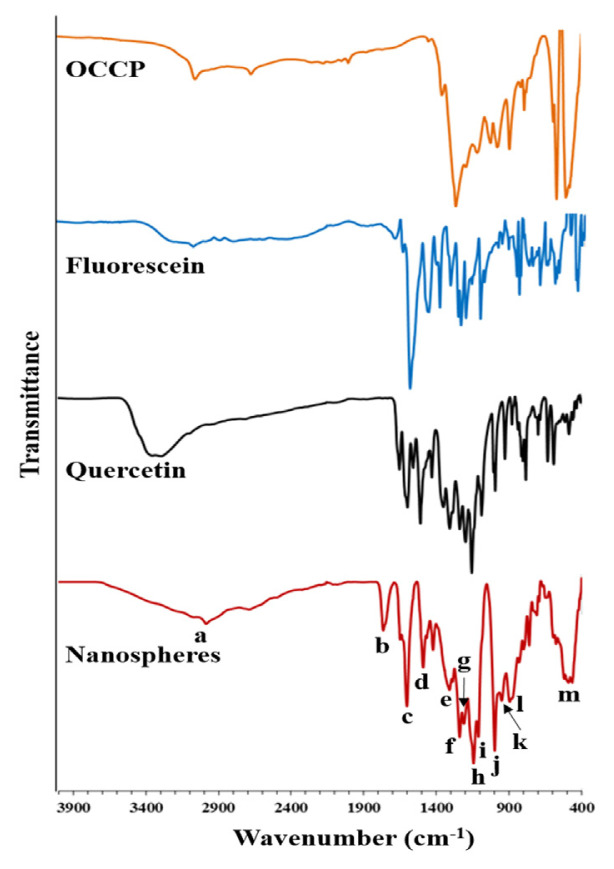
The FTIR spectra of the nanospheres, OCCP, fluorescein and quercetin.

The polyphosphazene nanospheres were characterised by FTIR spectroscopy by comparing with the FTIR spectra of quercetin, fluorescein and OCCP in Figure 4. Because of the highly crosslinked polymeric structure of the nanospheres, the wide band is observed between 3600 and 2400 cm^−^^1^ (a) that is corresponded to overlapped hydroxyl and aromatic C-H bonds. The peak at 1766 cm^−^^1^ (b) is attributed to the lactone ring (carbonyl group) of the fluorescein [74]. The carbonyl peak of quercetin is seen at 1603 cm^−^^1^ (c) [75]. The absorption at 1490 cm^−^^1^ (d) is corresponded to C-C stretching band. The peaks at 1307cm^−^^1^ (e) and 1144, 1110 cm^−^^1^ (h, i) are belonged to asymmetric stretching and symmetric stretching of C-O-C which indicate the presence of fluorescein and quercetin in the nanospheres. The P=N and P-N bonds of the OCCP rings are seen at 1240, 1208 cm^−^^1^ (f, g) and 952, 895 cm^−^^1^ (k, l), respectively. The sharp peak at 998 cm^−^^1^ (j) is attributed to the P-O-Ar that is showed the bonding between fluorescein, quercetin and OCCP. Besides, the peak at 490 cm^−^^1^ (m) is corresponded to P-Cl bond which indicates the nanospheres have a few amounts unreacted chloride atoms owing to steric hindrance. The all FTIR results demonstrated that quercetin and fluorescein were bonded to the OCCP to form the crosslinked structure.

**Figure 5:**
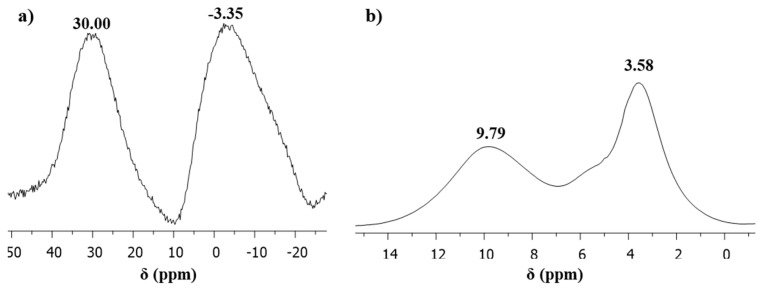
a) Solid-state ^31^P-NMR spectrum and b) solid-state ^1^H-NMR spectrum of the nanospheres.

To explain the substitution on OCCP and crosslinked structure of the synthesized nanospheres, solid-state NMR spectra (^31^P and ^1^H-NMR) were acquired (Figure 5). In the solid-state ^31^P-NMR spectrum of the nanospheres, two broad peak appeared at −3.35 and 30.00 ppm, indicating the presence of –N=P(–R_1_)_2_, –N=P(–R_2_)_2_, –N=P(–R_1_)(–R_2_) and –N=P(–Cl)_2_, –N=P(–R_1_)(–Cl), –N=P(–R_2_)(–Cl), respectively (Figure 5a). The spectrum signals are broad due to the overlapping of resonance signals of phosphorus atoms that have similar chemical environments. In addition, due to containing five hydroxyl groups of quercetin, binding in different combinations between quercetin and OCCP is possible. It is thought that the crosslinked structure is not fully substituted with fluorescein and quercetin because of the steric hindrance effects of the monomers. Therefore, the formed crosslinked structure is quite complex and difficult to completely explain [76]. Besides, the solid-state ^1^H-NMR spectrum can be seen in Figure 5b. In the solid-state ^1^H-NMR spectrum, two broad signals are seen at 9.79 and 3.58 ppm attributed to Ar-H and Ar-OH, respectively. It is seen that, some hydroxyl groups of fluorescein and quercetin could not react due to steric hindrance.

**Figure 6:**
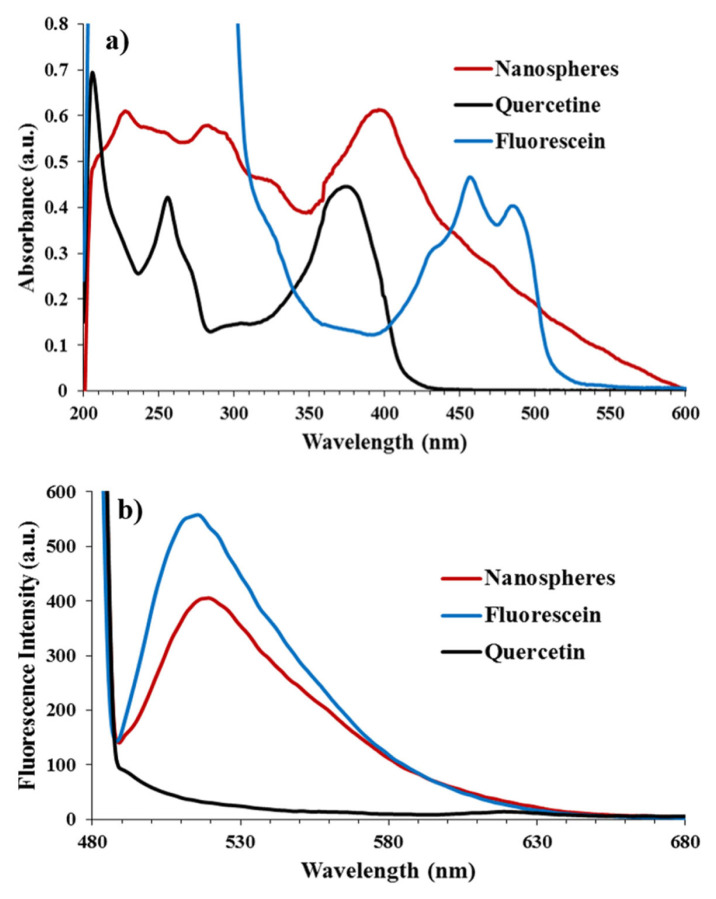
The UV-vis spectra a) and the fluorescence spectra b) of the nanosphers, fluorescein and quercetin in ethanol.

The UV-vis spectra of the nanospheres, quercetin and fluorescein were compared in ethanol (Figure 6a). The quercetin and fluorescein showed maximum absorption bands at 256, 374, 457, and 485 nm, respectively. The synthesized nanospheres exhibited wide absorption owing to their highly crosslinked structure and showed maximum absorptions at 228, 283, and 396 nm.

### 3.3. Fluorescence properties of nanospheres

The fluorescence emission spectra of the nanospheres, fluorescein and quercetin in ethanol were measured to compare their fluorescence behaviour (Figure 6b). The nanospheres and fluorescein exhibited strong emission peaks at 519 and 515 nm when excited at 470 nm, respectively. The fluorescence emission peak of the nanospheres is red-shifted probably because the connected fluorescein and quercetin changed the energy gap for the electron transition of fluorescein and quercetin in the nanospheres [46]. However, quercetin did not show fluorescence emission when excited at 470 nm. Besides, the OCCP rings are nonconjugated systems for electron transfer and are photochemically inert. Because, they have alternating P–N single and double bonds without any resonance [42, 77].

They isolate monomer moities in the nanospheres. Therefore, the electron and energy transfer between the monomer moities were effectively blocked [43].

**Figure 7:**
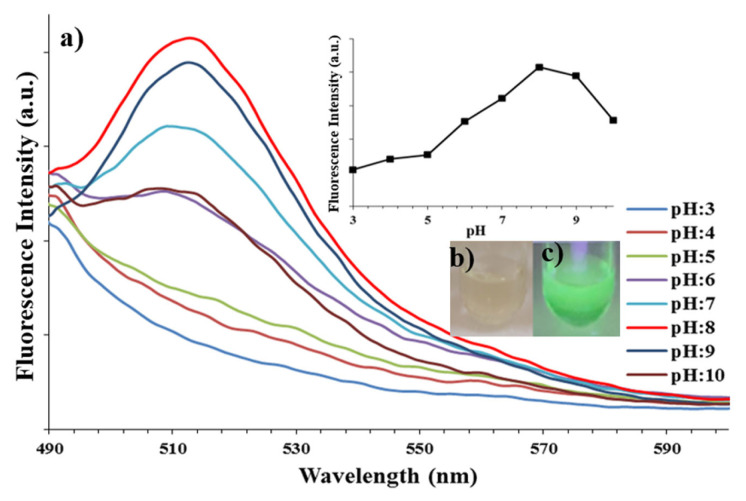
a) The fluorescence spectra of the nanospheres at different pH. The photographs of the nanospheres in pH 8.0 b) under daylight and c) UV 365 nm light.

The fluorescence emissions of aqueous dispersions of the nanospheres (5 mg mL^−^^1^) were measured at different pH between 3 and 10. It is seen that the fluorescence emissions of the nanospheres can change depending on pH in Figure 7a. The hydroxyl groups of the fluorescein and quercetin, and nitrogen atoms of the OCCP in the nanospheres have lone pair electrons. In basic medium, hydroxyl groups and nitrogen atoms can transfer electrons to fluorescein and quercetin moities. Therefore, the nanospheres exhibited higher fluorescence emissions at basic medium. However, in acidic medium, because of the protonation of hydroxyl groups and nitrogen atoms, the electron transfer is eliminated and the weakened fluorescence emission peaks are seen. Consequently, the optimum pH was determined as 8.0 [39]. Also, the photographs of the nanospheres in pH 8.0 under daylight and UV 365 nm light can be seen in Figures 7b and 7c as colourless and green, respectively.

**Figure 8:**
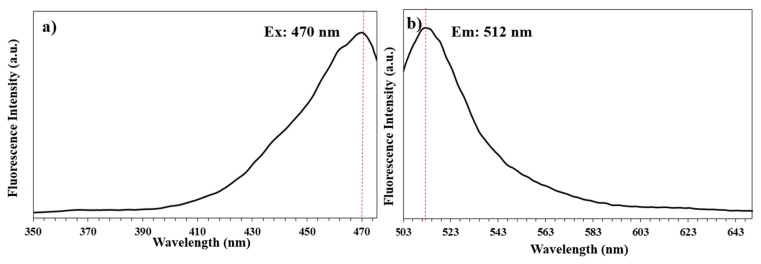
Excitation (a) and fluorescence emission (b) spectra of the nanospheres in pH 8.0.

In Figures 8a and 8b, the steady-state excitation and fluorescence emission spectra of the nanospheres in pH 8.0 at room temperature are showed, respectively. As can be seen, the maximum emission peak appears at 512 nm with a maximum excitation wavelength of 470 nm.

**Figure 9:**
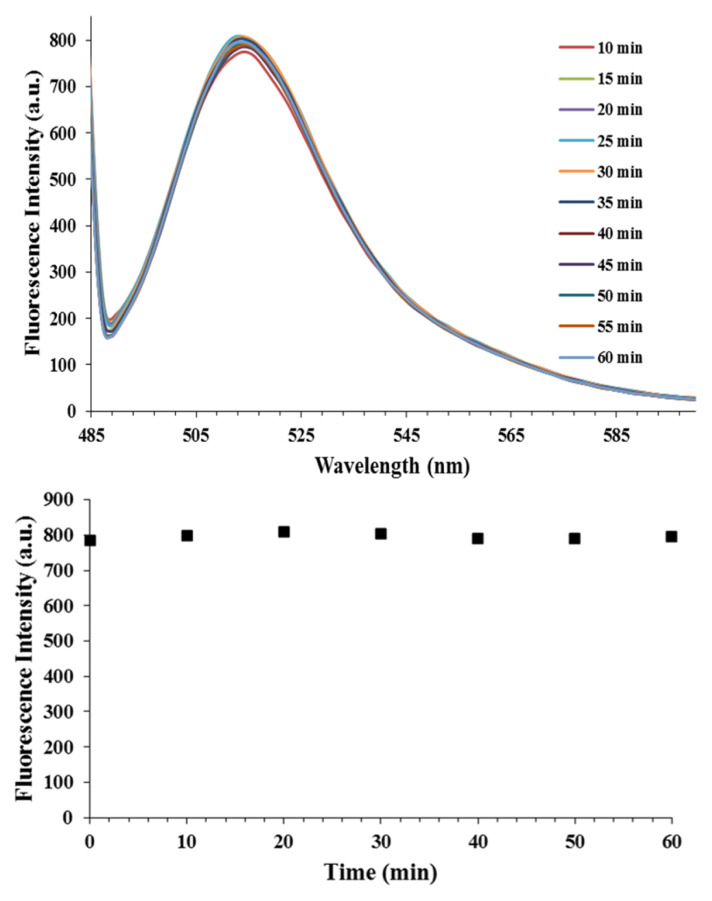
a) Fluorescence spectra of the nanospheres (30 mg mL^−^^1^ aqueous dispersion at pH: 8.0) with excitation wavelength at 470 nm at various irradiation times (0–60 min). (b) The fluorescence intensity of the nanospheres, versus UV-vis irradiation time (470 nm).

The photobleaching property of the synthesized nanospheres at pH: 8.0 was investigated. The nanospheres exhibited outstanding photobleaching stability under UV-vis irradiation at 470 nm during 60 min (Figures 9a and 9b). It was known that the photostability of fluorescein and quercetin is not good. However, the nanospheres gain excellent photobleaching property when they fastened to the OCCP. The highly crosslinked and organic-inorganic hybrid structure effectively retard photobleaching. The nanospheres have the potential to be used in sensor applications due to the photobleaching property.

**Figure 10:**
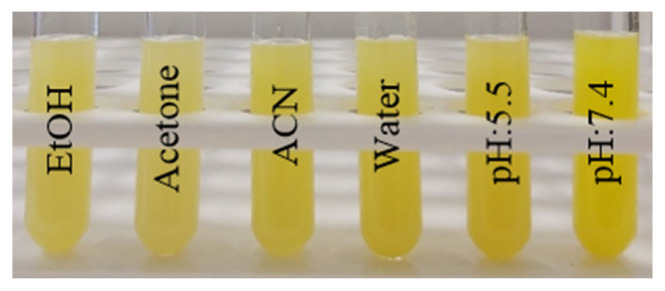
The photographs of the nanospheres dispersed in different solvents (1.0 mg mL^−^^1^), including ethanol, acetone, acetonitrile, water, pH: 5.5 and 7.4 buffer solutions.

Moreover, the photographs of the dispersions of the nanospheres in different solvents (1.0 mg mL^−^^1^) such as ethanol, acetone, acetonitrile, water, pH: 5.5 and 7.4 buffer solutions are presented in Figure 10. The nanospheres have excellent solvent resistance and dispersion ability in both organic and aqueous solvents due to the highly crosslinked and inorganic-organic hybrid structure.

## 4. Conclusion

In summary, the novel inorganic-organic hybrid, crosslinked and intrinsically fluorescent polyphosphazene nanospheres with an average diameter is 379 nm were successfully generated by self-assembly polycondensation polymerization. The fluorescein as an organic fluorescent dye and the anticancer drug, quercetin were reacted with OCCP to obtain the nanospheres. The one-pot synthesis procedure was easy and rapid. The obtained nanospheres were characterised by SEM, EDX, DLS, XRD, FTIR, solid-state ^31^P-NMR, solid-state ^1^H-NMR and UV-vis techniques. Quercetin was loaded to the fluorescent nanospheres covalently. The drug loading, DL (%) and entrapment efficiency, EE (%) of quercetin were calculated as 44.6% and 96.77%, respectively. Also, the fluorescence properties of the nanospheres were investigated. The optimum pH was defined as 8.0. The formation of a highly crosslinked structure led to enhancement of the outstanding optical properties, fluorescent intensity, photobleaching stability and solvent resistance. Hence, the nanospheres have a great potential both as a nanocarrier for delivery of quercetin, and as chemical or biological sensors, fluorescent labels.

Figure S1 The calibration curve obtained with standard quercetin solutions.

Figure S2 The self-assembly and formation mechanism of the nanospheres.

## Figures and Tables

**Table:** Comparison of reported results for drug loading and entrapment efficiency of quercetin.

Materials	DL (%)	EE (%)	Reference
Chitosan NPs	11.17	13.01	[66]
Lecithin-chitosan NPs	2.45	48.5	[67]
Micelles	8.2	45.3	[68]
Niosomes	1.65	94.67	[69]
Liposomes	-	72.5	[70]
Quinoa starch NPs	26.62	-	[71]
Halloysite-based carriers	1.96	-	[72]
Polyphosphazene nanospheres	44.60	96.77	This study
