# Supplementary figures and images for: Intrinsically fluorescent and quercetin loaded highly crosslinked polyphosphazene nanospheres: synthesis, characterization and fluorescence properties
Source: Turk J Chem. 2022 Apr 19;46(4):1269–80. doi: 10.55730/1300-0527.3433 (PMC10395785; doi:10.55730/1300-0527.3433)

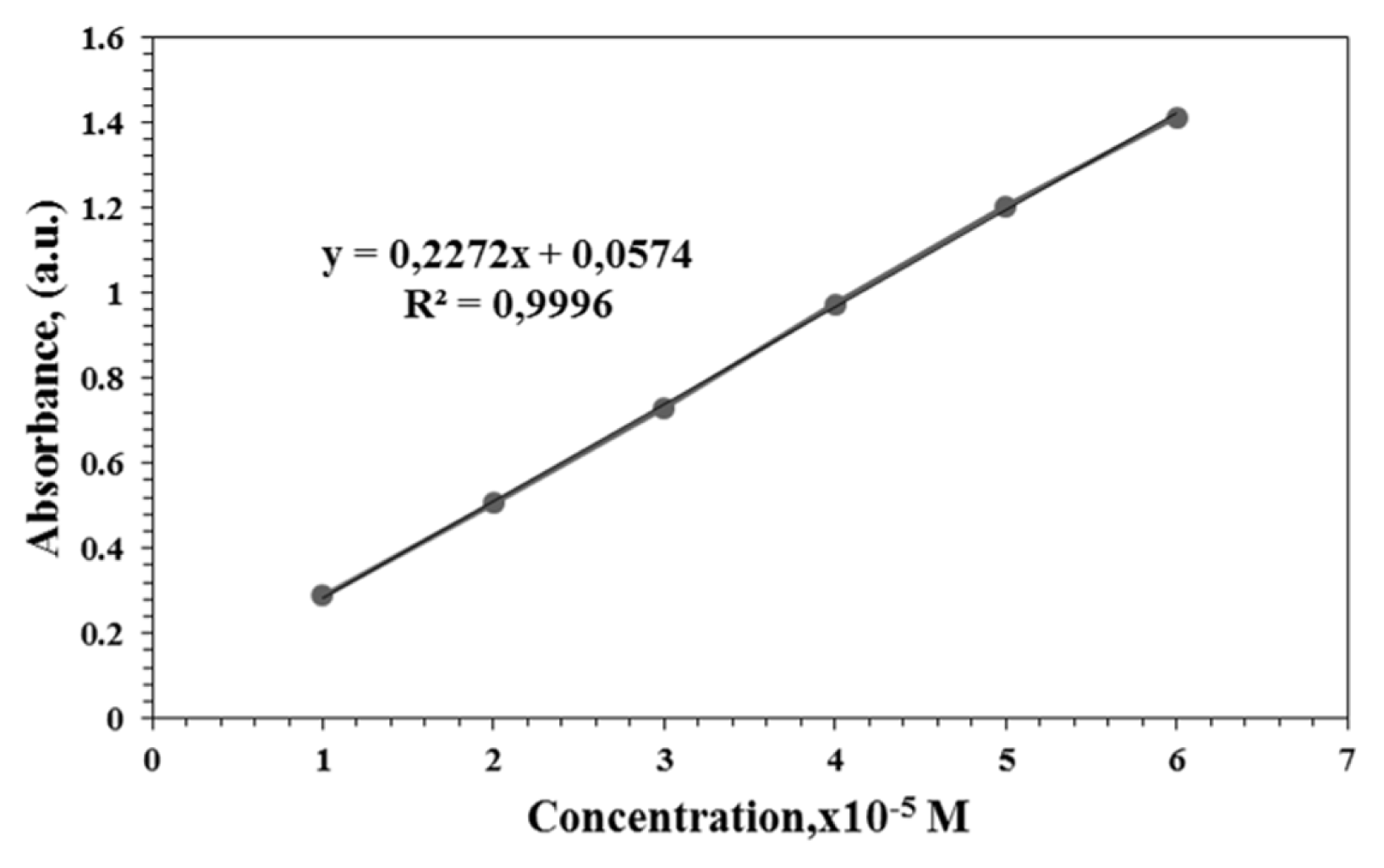

Supplement: Figure S1 — The calibration curve obtained with standard quercetin solutions. [file turkjchem-46-4-1269s1.tif]

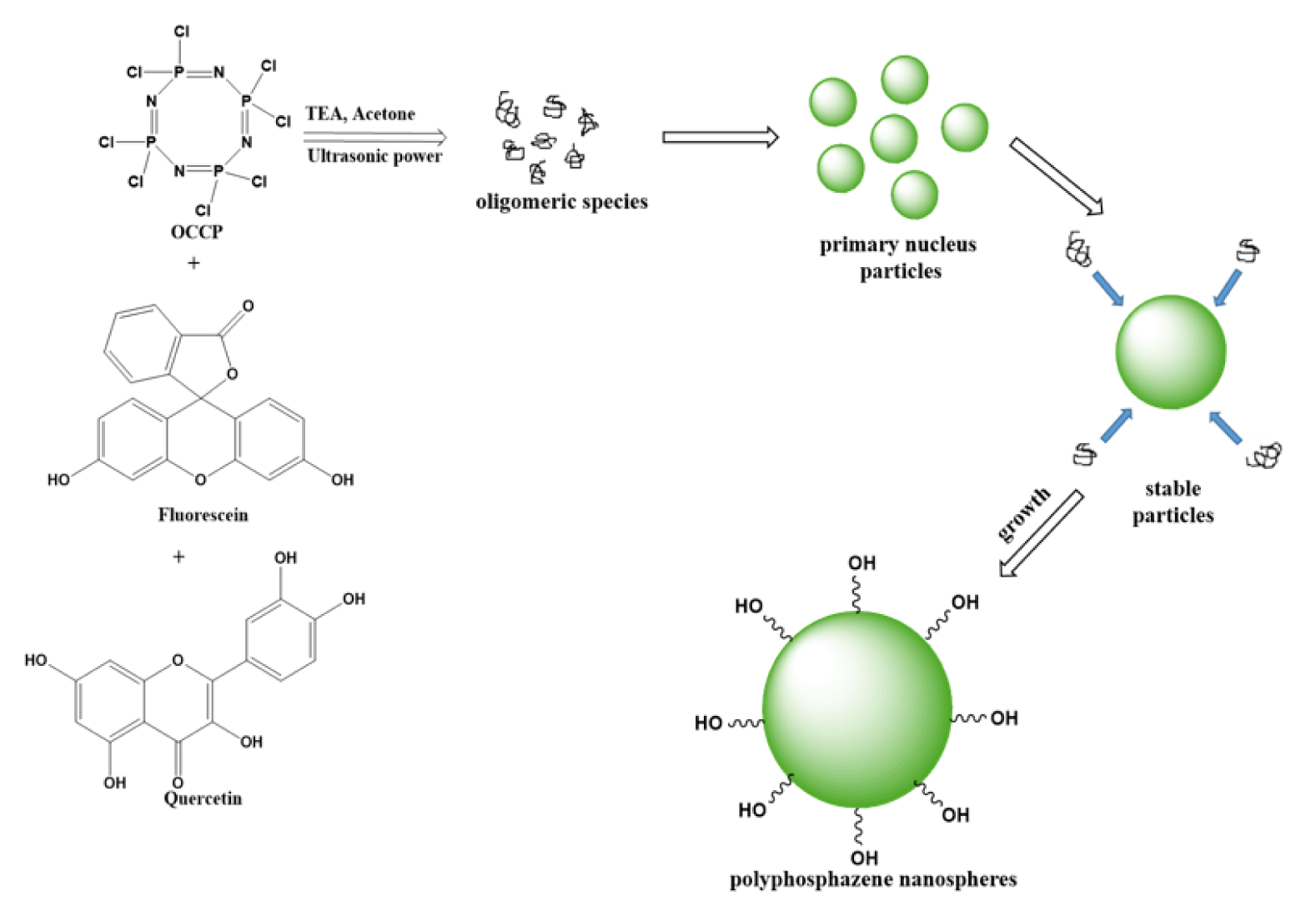

Supplement: Figure S2 — The self-assembly and formation mechanism of the nanospheres. [file turkjchem-46-4-1269s2.tif]
